# Healthcare providers’ attitudes towards care for men who have sex with men (MSM) in Malawi

**DOI:** 10.1186/s12913-019-4104-3

**Published:** 2019-05-17

**Authors:** Lester Kapanda, Vincent Jumbe, Chimaraoke Izugbara, Adamson S. Muula

**Affiliations:** 1 Department of Public Health, School of Public Health and Family Medicine, College of Medicine - University of Malawi, Blantyre, Malawi; 2 Department of Health Systems and Policy, School of Public Health and Family Medicine, College of Medicine, University of Malawi, Blantyre, Malawi; 3 International Center for Research on Women (ICRW), Washington DC, USA; 4 Africa Centre of Excellence in Public Health and Herbal Medicine (ACEPHEM) – College of Medicine, University of Malawi, Blantyre, Malawi; 5 Department of Mathematical Sciences, Chancellor College, University of Malawi, Zomba, Malawi

**Keywords:** Malawi, HIV prevention, Health care professions, Health professions students, Men who have sex with men (MSM), Sexual health services, Key populations

## Abstract

**Background:**

Men who have sex with men (MSM) are a priority group in Malawi’s national response to Human Immunodeficiency Virus (HIV) and Acquired Immunodeficiency Syndrome (AIDS). There are limited data on service providers’ acceptability to deliver appropriate sexual health services in relation to HIV prevention, care and treatment targeting the MSM. We assessed attitudes of healthcare providers already working, health professions students and faculty at health professions training institutions regarding the provision of MSM focused HIV related health services.

**Methods:**

We conducted a qualitative study between April and May 2017 in Lilongwe, Malawi. We purposively recruited 15 participants (5 health service providers, 5 health professions students and 5 faculty of tertiary health training institutions) among whom individual in-depth interviews were conducted. Interviews were audio recorded, transcribed and analysed thematically.

**Results:**

Participants recognized MSM as having health needs and rights. Participants generally expressed willingness to deliver appropriate healthcare because they perceived this as their professional responsibility. Participants suggested that it was the responsibility for MSM to disclose their sexual orientation and or preferences when they access care such that healthcare providers better anticipate their care needs. They suggested a need to increase the availability of MSM-centered and friendly health services as well as trained providers that are non-judgmental, non-discriminatory and have respect for people’s right to health care access.

**Conclusion:**

Despite widespread poor attitudes against MSM in Malawi, health service providers and health professions students and faculty accepted and were willing to provide MSM-focused health services. The acceptability and willingness of health service providers, health professions students and faculty to provide health services to MSM offer hope and scope for efforts to strengthen the delivery of health services and quality of care to MSM in Malawi.

## Background

In Malawi 1.8% of adult men between 20 to 39 years are estimated to be men who have sex with men (MSM) [1]. Men who have sex with men have a disproportionate HIV prevalence of 18.2% compared to 6.4% among the general population adult men [2–4]. Structural factors such as criminalization of same-sex, stigma and discrimination [3, 5, 6] are key barriers to targeted service availability and provision, access and utilization [7–10].

Perceived and experienced stigma and discrimination due to sexual preferences and confidentiality concerns in healthcare settings are among structural barriers to accessing services among MSM especially in settings where male to male sex is illegal [11, 12]. Men who have sex with men are at an increased risk for HIV [3, 13, 14] which is compounded by situations of low access to targeted health information and health services [7, 14–16].

In Malawi, MSM are among the key populations in relation to HIV acquisition and transmission [17, 18]. In addition to the recognition of MSM as a key population in HIV prevention, studies have documented the unmet health needs of MSM [19, 20]. Men who have sex with men need comprehensive HIV prevention services [10, 11].

Policy [17, 18] and published literature indicate the relevance of prioritizing MSM in HIV related healthcare [11, 21–23]. However, previous research in the African setting reported suboptimal uptake of services among MSM attributed to service providers who cannot be trusted and their non-responsiveness to the health needs of MSM in addition to enacted stigma [7, 9, 24–28].

Invisibility of MSM in healthcare and surveillance programs in Africa including Malawi warrant a need to explore attitudes of healthcare providers in relation to delivery of MSM focused HIV prevention, treatment and other related care. We sought to assess the healthcare providers’ attitudes and understand perceptions and suggestions related to strategies for enhancing delivery and use of appropriate and targeted HIV related healthcare among MSM.

## Methods

### Study design and approach

The study was qualitative in nature. In-depth interviews were used to assess attitude to deliver sexual healthcare in relation to HIV and AIDS services to MSM among health service providers, health professions students and faculty of health professions training institutions.

### Study setting

The study was conducted at purposively selected sites. These were: two public health professions training institutions, one Christian Health Association of Malawi (CHAM) health facility (which also trains health professionals) and three public primary health facilities in Lilongwe, Malawi. We decided to conduct this study in Lilongwe the capital city which registered second highest estimate of MSM in a previous study [1]. Secondly, Lilongwe has over three health professions training institutions all headquartered there and the primary health facilities within it would provide the needed primary information on health care delivery appropriate and targeting MSM as per the objective of this study.

### Sampling

We approached and recruited 15 participants for in depth interviews through purposive sampling technique [29]. We selected health professions cadres who were likely to provide primary research data on willingness to provide MSM targeted HIV related health services. Potential participants were eligible if they were at least 18 years old, had more than 1 year health services work experience or were in the final year of health professions training and able to provide informed consent in either English or Chichewa (local language).

In a related study on health service seeking and provision practices among MSM and health service providers in Malawi, saturation was reached by the fifth participant and enrollment was stopped [25]. In our study, we reached saturation by the twelfth participant. However, we went ahead to recruit thee more to ensure that indeed no new information was coming out.

### Selection of in-depth interview participants

Participants comprised clinical officers and nurses in practice, and final year nursing and clinical officer students. We expected that some healthcare professionals would report that no MSM healthcare training was provided to them during pre-and in-service training, therefore faculty members from institutions which train health professions were included to validate such data. Consideration was also made to assess whether the currently trained health professions students are being prepared on the provision of appropriate sexual health care targeting MSM. As one way of ensuring maximum variation, we managed to elicit views of diverse categories of healthcare professionals with different ages, sex, and years of occupation experience.

### Data collection

Three interview guides one for health service providers already working, the second for final year health professions students and finally for faculty were made in accordance with the study objectives. The questions were open ended, with probes used to explore points raised by interviewees or for clarification where more information was required. Trained research assistants collected data through in-depth interviews after obtaining informed consent from 15 participants selected from all the participating sites.

The interview guides were used depending on language preference of the interviewee (English or Chichewa). All participants preferred to be interviewed in English language. We collected demographic data, personal views on men who have sex with men and suggestions towards provision of sexual healthcare targeting and appropriate for MSM. The average time taken for each interview was 40 min. We allowed enough time with participants to ensure that adequate data was collected during the interview. Participants were free to ask questions on issues they felt were not clear to them. Permission was obtained to audio record the interviews. Interviewers were ready to take notes where an audio recording would have been declined. Beside the 15 participants, two additional health professions’ faculty requested not to proceed with interview sessions as they expressed that it was against their religious beliefs to talk about MSM.

### Data management and analysis

Recorded interviews were transcribed verbatim. We reviewed the transcribed data to ensure understanding and then compared these (transcripts) with the original audio-recordings for accuracy. The primary author read and reviewed all the transcripts multiple times and selected one transcript for the initial open coding. To validate the coding process, a clean copy of the same transcript was de-identified and given to another experienced qualitative researcher to conduct a separate independent open coding which were later verified by co-authors. The primary author assessed both coding outputs and came up with one generic coding frame for indexing the rest of the codes. Relationships and comparisons between themes were generated from the coding frame in an iterative process. This ensured that attention was given for consistent patterns within the data focusing on similarities and differences on responses given by participants to aid analysis and interpretation. We generated themes inductively and deductively. Our approach to data analysis was based on thematic analysis in line with the study aim [30, 31].

### Trustworthiness

Four measures were taken to consolidate the study’s trustworthiness [32]. Before the beginning of data collection, a meeting was held with MSM community representatives, healthcare professionals in order to understand the culture related to health service provision which was appropriate to, and targeting MSM. To enhance credibility, we triangulated the data across the three data sources (health service providers already in service, health professions’ students and faculty). We ensured study’s dependability and confirmability by keeping a record of each study step and by identifying and providing exemplar quotations included in this paper. Adequate description has been provided in the limitations regarding transferability of the findings.

## Results

### Socio-demographic characteristics of the study participants

Fifteen participants were recruited into the study. Their ages ranged from 19 to 48 years (see Table 1 for details).

**Table 1:** Basic demographic characteristic of participants

Participant	Age	Gender	Years of service	Religion	Designation
HSP 1	28	Female	2	Christian	Clinical
HSP 2	47	Male	10	Christian	Clinical
HSP 3	33	Male	4	Christian	Clinical
HSP 4	38	Female	12	Christian	Nursing
HSP 5	26	Male	3	Christian	Clinical
Lecturer 1	40	Female	7	Christian	Nursing
Lecturer 2	48	Male	15	Christian	Clinical
Lecturer 3	39	Female	3	Christian	Nursing
Lecturer 4	35	Male	2	Christian	Clinical
Lecturer 5	43	Female	9	Christian	Nursing
Student 1	32	Male	Final year student	Christian	Clinical
Student 2	23	Female	Final year student	Christian	Nursing
Student 3	32	Female	Final year student	Christian	Nursing
Student 4	19	Male	Final year student	Christian	Clinical
Student 5	24	Female	Final year student	Christian	Nursing

*HSP* Health service provider already working

### Acceptability to provide sexual health services targeting MSM

Most participants considered it acceptable to provide MSM with their required and targeted health services in relation to HIV prevention, treatment and care. Explanations for acceptability emphasized six major issues namely:

(i) Perceived role and responsibility to provide healthcare: participants expressed willingness to deliver appropriate healthcare because they perceived themselves as having a role and responsibility towards the health needs of MSM. Participants narrated that health service providers have a role to play; and cannot choose who to assist and not to assist. Participants also expressed that health service providers do not need to show bias when providing health care to people. They ought to be equitable when attending to health needs of MSM.

“….*it is our responsibility as healthcare professionals to provide healthcare to people despite what they do in life, so not helping people like men who have sex with men whenever they seek care means that we are not doing our job adequately*”.**[Student 3].**

(ii) Respect for equality of humans**:** the humanity of MSM was reported and appreciated. Participants accepted to provide health services to MSM considering that MSM are just like any other human being with feelings and concerns. Additionally, MSM are part of the society and the only difference was that they have different sexual behaviours from that which the general society regards as normative. Participants further expressed that the MSM community is a sub-population which must be understood in the country and they equally require services just as others.

“*Well, aaah….my view is that, men who have sex with men are just like any other human being, they are normal people just like others. They do have feelings what so ever just like any other human being”* **[Lecturer 4]**

*“Aaaah.., whenever I hear about men who have sex with men, I take them as any other normal people who may as well need every kind of help like every person does* ...” **[Health Service Provider (HSP) 3]**

(iii) Universality of health needs: the participants reported that MSM need to be provided with health services in light of the view that they have health needs like the rest and ought to have equal access to health and wellness and therefore were expected to access healthcare. The participants gave advice that whenever MSM approach health service providers seeking healthcare, they must be assisted accordingly and the following statement summarizes the point of view:

“*Well, aaah as I mentioned earlier on that men in the same sex practice are just like any human being in any way, they also need health care just like others, for example, whenever they are not feeling well, they are supposed to come to the hospital and get treated, just like any other human being would do”*. **[Lecturer 4]**

(iv) Conformity to MSM rights to healthcare access: participants felt that MSM have the right to access healthcare like all others. In this regard, the provision of health services to MSM was in conformity to the healthcare access right that MSM have. Alongside the right to healthcare access, participants further reported that MSM are equal and legitimate users of healthcare services. Participants further expressed that the health needs required by MSM should not be overlooked in the healthcare settings and what must be done is to help MSM whenever they have come to seek health services as expressed by the statement:

“*Since each and every person has got a right to access health services so MSM too deserve access to such care. In addition, as health service providers, we do help patients as per their human and health rights and in the case MSM they are like any other person, they have got a right to access treatment, to care and support like any other person*”. **[Student 5]**

(v) Disclosure of sexual behaviours**:** participants expressed willingness to provide targeted healthcare to MSM by proposing that as MSM interact with health service providers, it is in their best interest to disclose their sexual practices as this would be an entry point for a health service provider to learn more about the individual, note and record detailed information during medical consultation. Participants further expressed that disclosure would assist in risk factor analysis for the MSM which may then facilitate provision of appropriate and targeted healthcare. Overall, participants were also of the view that where MSM are able to disclose their sexual preferences and practices, such would enhance patient-provider communication which may translate into MSM receiving appropriate and quality care as expressed in the statement below:

*“I think it is important for MSM to report their sexual lifestyles and practices in the first place, in fact it may facilitate the quality of care one ought to receive. Sometimes the sexual behaviours of an individual can be over looked, then the actual health problem can be missed and can lead to negative consequences in future*”. **[HSP 3]**

(vi) Perceived elevated HIV risk among MSM: participants expressed willingness to provide MSM targeted health services considering that MSM are at higher than general population risk of HIV. Beside HIV, there were also concerns with regards to other sexually transmitted infections (STIs) for instance, syphilis.

*“Men who have sex with men need to realise that those using the anus for sex are more susceptible to HIV and other sexually transmitted infections. In the presence of HIV and other STIs, the chances of a person acquiring and transmitting such infections are more compared to using the vagina for sex.”* **[Lecturer 5]**

Addressing MSM health needs has benefits to community members: considering that MSM are at higher than usual risk for HIV acquisition and transmission, participants recognised that supporting the heath needs of MSM was inevitable. Consequently attending to MSM specific health needs would as well advantage other members of the community who may have sexual connections or share sexual partners with MSM:

*“…..in fact another comment might be that, there is a lot to be done, more especially on the healthcare targeting MSM as I tend to believe that they might be the potential sources of some communicable infections such as sexually transmitted infections to the community”.* **[HSP 3]**

### Perspectives on how to improve the delivery of care for HIV prevention, treatment and care targeting MSM

In line with acceptability to provide MSM with appropriate and targeted health services, participants in this study expressed additional ideas with the view of bridging the current gaps in the service delivery through the following three areas:

Availability of patient centered care: participants proposed ways where patient centered healthcare may be provided to MSM. They reported that sexual healthcare should be directed to the whole person without discrimination, but in response to the needed healthcare. Participants encouraged openness on the part of MSM to disclosure sexual practices as this would enhance proper history taking and patient screening in order to provide appropriate management.

*“We need to help MSM when they come to seek health care and also when they have revealed their sexual preferences and behaviours to us as health service providers. We have to try to deal with the health problem that they may have brought forward. As a rule, when a patient comes, you handle them according to their problem. Proper history and examination would assist provide appropriate care”.* [**HSP 1**]

The availability of non-judgmental healthcare*:* Participants expressed that non-judgmental healthcare should be provided to MSM whenever they seek healthcare:

*“…..when men who have sex with men have revealed themselves to us as health service providers, we need not to judge them but instead we have to try to deal with the problem that they may have presented with…….”* [**HSP 2**]

Availability of nondiscriminatory healthcare: participants also highlighted that MSM must not be discriminated against in the healthcare settings as expressed below:

“*Men who have sex with men should not be segregated or discriminated against. As health service providers we have a duty to serve humanity without discrimination”* **[Student 4]**

## Discussion

We assessed health service professionals in practice, health professional students and faculty members’ attitudes in relation to provision of HIV related health services to MSM in Malawi. Research in Africa, has highlighted on a number of challenges that MSM experience in accessing quality health services including unfavorable patient-provider relationships [33–35]. We aimed to identify opportunities for making HIV-related health services accessible to MSM as a key population in the national response to HIV and AIDS despite the prevailing same-sex legal restrictive and culturally sensitive environment in the country [9, 36].

In this study participants accepted and were generally supportive to the provision of MSM-focused health services. They expressed that MSM have health needs and equal status like others in accessing appropriate healthcare. We do not however, wish to minimize current barriers as often there is in the country, stigma and discrimination towards MSM and denial of their existence as sexual minority and a key population in HIV response [10, 21]. Recognition of MSM as having health needs and equal status in accessing healthcare was possible in Kenya and South Africa following sensitization training on MSM health needs [37, 38]. Our finding, is in contrast to prior research in most African countries where MSM’s acceptance as equal citizens with full entitlement to health services and prevention information access, is challenging [39]. However, recognition of MSM as equal citizens requiring healthcare as reported in our study is positive in that if harnessed and services developed and offered, service barriers may not be as severe as it has been previously.

Participants reflected on their professional responsibility that calls for enactment of equity and respect when providing care without imposing personal values on the men. This finding agrees with research from Kenya where health service providers highlighted the relevance of their health professional role in attending to people’s health concerns as demanded by their jurisdiction and to the best of their capability. They said that they have a professional duty and societal obligation to provide health services to MSM without imposing segregation [38]. Provision of MSM focused healthcare requires no inclination to personal judgement and discriminatory attitudes to MSM in order to be effective in delivering appropriate and targeted HIV related healthcare as reported in Kenya and South Africa [37, 38].

Health service providers as privileged and strategically placed members, particularly given their respected position in society, are influential to the MSM health needs and delivery of such healthcare [40]. On this, literature indicates that society power can affect delivery of appropriate healthcare [41]. The mindset change on supporting MSM healthcare provision demonstrated in our study, agrees with previous research from Swaziland [42]. Similar positive attitude on MSM health needs has also been displayed by healthcare providers as reported in Tanzanian research [43]. Such positive changes towards MSM health service provision as highlighted and reported in our study and the two studies above must be appreciated as the situation may facilitate equitable healthcare access as well as improved MSM-provider relationships [44].

Our study also highlighted universality of health needs, MSM rights to healthcare access, equal and legitimate use of healthcare among MSM. This is in line with rights-based approach to ensure access to services of the highest possible quality [44]. It is a finding in line with the guidelines for best practices in providing care to MSM that World Health Organization (WHO) has outlined and encourages adoption in maximizing HIV and AIDS response [45]. The same finding also agrees with research from Ghana where healthcare providers generally supported the attitude that MSM’s rights need to be respected, which included access to professional health care [27].

Participants expressed concern about MSM being at an increased risk for HIV acquisition and transmission, which is consistent with previous findings [1, 46, 47] and that preventive services be made a priority to them as a key population. In line with provision of HIV preventive services, participants expressed that attending to MSM specific sexual healthcare has perceived benefits to community members who share sexual partners with MSM. This finding agrees with research in Africa reporting that HIV amongst MSM remains a priority public health concern [3, 48]. However, Beyrer and others stated in their 2014 published article that interventions tailored towards MSM have great impact on HIV prevention among them as well as the general population [49].

Patient-centered healthcare and availability of friendly services through providers that are nondiscriminatory and non-judgmental were other proposed strategies suggested if MSM are to receive responsive healthcare. Patient-centered healthcare is a strategy described as responsive to patient’s needs and the circumstances they find themselves in [50]. It is an appropriate strategy considering that MSM report differential gender expressions, identities and sexual practices that require targeted interventions than group interventions [26, 37]. This finding echoes that from qualitative research among healthcare providers in Kenya, although the study was implemented after providers had received sensitivity training on MSM healthcare provision [40]. Patient centered healthcare strategy is of great importance considering that stigma and discrimination disadvantage a vulnerable group (MSM) [51, 52].

Men who have sex with men are continuously being underserved in terms of appropriate healthcare due to non-existence of MSM friendly health services in sub Saharan Africa, Malawi inclusive [10, 12, 53]. The situation is exacerbated by inadequately trained healthcare professionals who have negative attitude to provide respectful MSM centered healthcare [40]. Additionally, inadequate investment dedicated to MSM health as a result of not prioritizing their health [54] and the socio-political environment that continues to resist same-sex relationships also add to the underserving of MSM [55]. The impact of not having MSM friendly health services is that it influences negative MSM health seeking behaviours and leads to late presentation for care [3, 9], ineffective healthcare provision as well as negative health outcomes. At the moment center for the development of people (CEDEP), a sexual minority human rights watch dog is working with eighty public and private health facilities in eleven districts in Malawi. It has to date trained two hundred and fifty-eight health service providers on MSM friendly health service provision and also established drop in centers (DICs). The DICs serve as stand-alone clinics where, in addition to recreation and getting reproductive health information, MSM clients can test for HIV, screen and get treated for sexually transmitted infections (STIs) [56]. This development will increase visibility of trusted and competent health service providers who can be supportive of MSM needed services.

Disclosure fears among MSM and service providers in relation to the provision of services to men in same-sex sexual relationships have been previously highlighted [25]. Additionally, previous research has underscored MSM’s inadequate preference to disclose their sexual orientation and other health-related behaviors to facilitate appropriate clinical decisions [57]. Healthcare providers reported that it was important for MSM to identify themselves to health service providers for appropriate healthcare although other participants were of the contrary view. Participants proposed that MSM would be assisted appropriately at health facilities if they disclosed their sexual behaviours. This proposal agrees with recommendation that has seen a manual on MSM’s health needs training guide for front-line healthcare providers in Africa revised to add a module on sexual identity and coming out among MSM. This development followed systematic review on emerging themes for sensitivity training modules of African health service providers attending to MSM [58]. Literature states that sexual orientation, behaviours and gender identity are social determinants of health among sexual minorities [59]. However, there is need to explore what MSM themselves think about the proposed preference to disclose their sexual orientation and other health-related behaviors. Without the necessary safeguards for confidentiality and security, such a recommendation may be premature.

### Study limitations

Our study is subject to some limitations. It targeted selected health professions schools and health facilities within Lilongwe city. Secondly we only managed to elicit views of participants from the Christian faith not by design, although religion was not an inclusion criterion for this study. Findings may therefore only apply to the context in which data were obtained. Views expressed in these findings reflect those of the few healthcare providers and students interviewed and raise important questions regarding the transferability of the findings to the entire health professionals community in Malawi. It is worth noting that qualitative studies do not attempt to generalise findings. However, the description of acceptability to provide HIV related health services among health service providers already in service, final year health professions students and faculty as elicited in this study could be valid in another setting with similar context.

There were variations in probing from the research staff who facilitated interviews as shown by the inadequate probing in eliciting cultural and religious beliefs of prospective participants who declined study participation. Despite the stated short comings, our study provides new insight on acceptability to provide targeted and appropriate health services for MSM among health professions in Malawi.

## Conclusion

Healthcare professionals in practice, health professions students and faculty members were willing to provide MSM focused HIV related health services in Malawi. They recognised and appreciated MSM like others that have equal and legitimate service, health needs and healthcare access rights. Participants proposed strategies for MSM to receive specific health care despite MSM restrictive laws in Malawi. This research make available a basis for planning and implementation of innovative MSM focused interventions. Strengthening these findings could facilitate access to quality health services, finding a middle ground for client-provider relationships and may reduce delays or avoidance of HIV services use by MSM. More research is required to capture more views and suggestions from the wider health service provider community and understand extra proposed strategies of providing appropriate and targeted MSM health care in Malawi.

## Abbreviations

ACEPHEM: Africa Centre of Excellence in Public Health and Herbal Medicine
AIDS: Acquired Immunodeficiency Syndrome
CARTA: Consortium for Advanced Research Training in Africa
CEDEP: Centre for the Development of People
CHAM: Christian Health Association in Malawi
COMREC: College of Medicine Research and Ethics Committee
DFID: Department for International Development
DIC: Drop In Centres
EHPSA: Evidence for HIV Prevention in Southern Africa
HIV: Human Immunodeficiency Virus
HSP: Health service Provider
ICRW: International Center for Research on Women
MSM: Men who have Sex with Men
SRHR: Sexual and Reproductive Health and Rights
STI: Sexually Transmitted Infections
